# Germline HAVCR2/TIM-3 Checkpoint Inhibitor Receptor Deficiency in Recurrent Autoinflammatory Myocarditis

**DOI:** 10.1007/s10875-024-01685-x

**Published:** 2024-03-15

**Authors:** Nora Pernaa, Anni Vakkuri, Miika Arvonen, Outi Kuismin, Wenny Santaniemi, Virpi Glumoff, Elisa Lappi-Blanco, Ulla Lantto, Marjo Okkonen, Kari Kaikkonen, Juhani Junttila, Risto Kerkelä, Pirjo Åström, Timo Hautala

**Affiliations:** 1 Research Unit of Biomedicine and Internal Medicine, Medical Research Center Oulu, University of Oulu and Oulu University Hospital, Oulu, FIN-90014 Finland; 2 Department of Pediatrics, Kuopio University Hospital and University of Eastern Finland, Kuopio, Finland; 3 Department of Clinical Genetics, Oulu University Hospital, Kajaanintie 50, Oulu, 90220 Finland; 4 Medical Research Laboratory Unit, Faculty of Medicine, University of Oulu, Oulu, Finland; 5 Department of Pathology, University of Oulu, Aapistie 5, Oulu, Finland; 6 Department of Otorhinolaryngology-Head and Neck Surgery, Oulu University Hospital, Oulu, Finland; 7 Biocenter Oulu, University of Oulu, Oulu, Finland; 8 Infectious Diseases, Oulu University Hospital, Oulu, Finland

**Keywords:** Myocarditis, Inborn error in immunity, Autoinflammation, Interleukin-1, T-lymphocytes

## Abstract

Myocarditis can be caused by viral infection, drug reaction or general inflammatory condition. To provide understanding on inflammatory myocarditis, we describe clinical, genetic, and immunological properties of a young male patient who suffered from recurrent myocarditis episodes since the age of four years. Electrocardiography, troponin I/T, echocardiography, myocardial magnetic resonance imaging and histological findings were consistent with recurrent myocarditis episodes. Homozygous c.245 A > G p.Tyr82Cys pathogenic variant in Hepatitis A Virus Cellular Receptor 2 (*HAVCR2*) gene encoding T cell immunoglobulin and mucin domain-containing protein 3 (TIM-3) receptor was found. Peripheral blood mononuclear cells were collected when the patient was asymptomatic; CD4^+^ and CD8^+^ T lymphoblasts, CD56^+^ natural killer cells and CD14^+^ monocytes were negative for surface TIM-3 expression. In vitro, TLR4 mediated interleukin-1β (IL-1β) response was high after LPS/ATP stimulation. Clinical symptoms responded to IL-1 receptor antagonist anakinra. TIM-3 p.Tyr82Cys CD4^+^ and CD8^+^ T cell proliferation in vitro was unrestrained. Findings on IL-2, interferon gamma, regulatory T cells, signal transducer and activator of transcription (STAT) 1, 3 and 4 phosphorylation, and PD-1 and LAG-3 checkpoint inhibitor receptor analyses were comparable to controls. We conclude that TIM-3 deficiency due to homozygous *HAVCR2* c.245 A > G p.Tyr82Cys pathogenic variant in the patient described here is associated with autoinflammatory symptoms limited to early onset recurrent febrile myocarditis. Excessive IL-1β production and defective regulation of T cell proliferation may contribute to this clinical condition responsive to anakinra treatment.

**Supplementary Information:**

The online version contains supplementary material available at 10.1007/s10875-024-01685-x.

## Introduction

T cell immunoglobulin and mucin-domain containing-3 (TIM-3) is a checkpoint inhibitor receptor encoded by the hepatitis A virus cellular receptor 2 (*HAVCR2*) gene [1]. This type I transmembrane receptor was initially identified as a marker for interferon gamma (IFN-γ) secreting T cells [2]. TIM-3 mediates inhibitory and activating signals, with an important role in infection, autoimmunity, inflammation, peripheral tolerance, and anti-tumor immunity [1]. In an experimental mouse myocarditis model, blocking of TIM-3 signaling increased inflammation at least partly due to regulatory T cell (Treg) mediated mechanisms [3, 4]. In humans, germline autosomal recessive TIM-3 deficiency (p.Tyr82Cys, p.Ile97Met) predisposes to rare subcutaneous panniculitis-like T cell lymphoma (SPTCL) complicated by hemophagocytic lymphohistiocytosis (HLH) [5]. A case of primary HLH without lymphoma has also been described in a biallelic (p.Ile97Met and p.Thr101Ile) TIM-3 deficient patient [6]. High production of interleukin-1β (IL-1β), tumor necrosis factor alpha (TNF-α) and IFN-γ was demonstrated in vitro in peripheral blood cells of SPTCL/HLH patients [5, 7]. However, early onset autoinflammation in a TIM-3 deficient patient in absence of malignancy or HLH has not been previously described.

Myocarditis in humans with an incidence ranging from 1 to 10 cases per 100,000 persons per year, can be caused by viral infection, immune-system activation or exposure to toxins or drugs [8]. Adenoviruses, enteroviruses, parvovirus B19, cytomegalovirus and coronaviruses, for example, are thought to be among the most common causative infectious agents [9]. Exposure to pharmacological agents such as antipsychotic agents, cytotoxic drugs, salicylates, and vaccines as well as immune checkpoint inhibitor (ICI) treatment may predispose to myocarditis [10]. Although most patients recover from myocarditis, *MEFV* mutations in familial mediterranean fever (FMF) or *STAT1* gain-of-function mutations, for example, may contribute to myocardial injury due to aberrant host immunity [11–15]. However, genetic and biological mechanisms of autoinflammatory myocarditis remain poorly understood. In this case report, we describe immunological features in a TIM-3 deficient patient suffering from early onset autoinflammatory myocarditis episodes.

## Materials and Methods

### Patient

The patient provided written informed consent to participate in this research in accordance with the Declaration of Helsinki.

### Whole Exome Sequencing

Genomic DNA was extracted from the patient’s blood sample and used for whole exome sequencing (WES) at Blueprint Genetics (Helsinki, Finland) (https://blueprintgenetics.com/tests/whole-exome-sequencing).

### Immunohistochemistry

Endomyocardial biopsies were collected during myocarditis episode. Samples were fixed in phosphate-buffered 10% formalin (pH 7.0) and embedded in paraffin. The samples were deparaffinized, boiled in Tris-EDTA buffer for 30 min and incubated with Dako REAL Peroxidase-Blocking Solution (Agilent technologies; S2023) for 30 min. The sections were incubated with TIM-3 antibody 1:50 (R&D Systems; MAB23652) for 1-hour at room temperature (RT). After washing, a Dako REAL anti‑rabbit/mouse secondary antibody (Agilent technologies; K500711-2) was added and incubated for 30 min at RT. Target antigens were visualized by using Dako REAL DAB + Chromogen (Agilent technologies; DAKO K500711‐2) for 90 s. Meyer’s Hematoxylin (Sigma-Aldrich; MHS32) was used for counterstaining. Nikon Eclipse 50i microscope equipped with a Nikon DS-Fi3 camera was used for imaging.

### Isolation and Culture of Peripheral Blood Mononuclear Cells

Peripheral blood mononuclear cells (PBMCs) from the patient and healthy sex-matched controls were isolated by Ficoll-Paque gradient centrifugation (lithium heparin tubes). The cells were aliquoted in 90% FBS (ThermoScientific; SV301800.03) and 10% dimethyl sulfoxide (DMSO) (Applichem; A3672,0250) and stored at -140 °C. Cells were cultured in RPMI 1640 (Sigma Aldrich; R0883), supplemented with 100U penicillin and 100 µg/ml streptomycin (Sigma Aldrich; P0781), 10mM HEPES (Sigma Aldrich; H0887), 2mM L-glutamine (Sigma Aldrich; G7513) and 10% FBS (ThermoScientific; SV301800.03) at 37℃ with 5% CO_2_ in a humidified incubator.

### Inflammasome Activity Assay

PBMCs from patient and healthy controls were plated at 1.5 × 10^6^/ml density in the conditions described above. The next day, 1 µg/ml lipopolysaccharide (LPS) (Sigma Aldrich; L3012) was applied to the cells for 6 hours followed by an additional 45 min with 5mM ATP (Sigma Aldrich; A6419). The cells were pelleted, and the medium was stored at -80 °C. IL-1β was measured using an enzyme-linked immunosorbent assay (ELISA) (R&D Systems; DY201) according to manufacturer’s instructions. The experiment was performed two times with two healthy controls, all samples were analyzed in duplicates.

### Flow Cytometry

In all experiments, PBMCs from the patient and two healthy sex-matched controls were used unless stated otherwise. All flow cytometry samples were analyzed in duplicates unless stated otherwise. The cells were cultured as described above. Phosphate buffered saline (PBS) (Sigma Aldrich; D1408) with 2% FBS (ThermoScientific; SV301800.03) was used for washing, unless stated otherwise. The dead and alive cells were stained with a LIVE/DEAD™ Fixable Near-IR Dead Cell Stain Kit (Invitrogen; L10119) according to manufacturer’s instructions. All antibodies used in the flow cytometry are listed in the supplementary materials. The data was collected with BD LSRFortessa™ using BD FACSDiva software and analyzed with FlowJo™ 10 (BD Biosciences).

#### Cell Surface and Intracellular Staining of TIM-3 in NK Cells and Monocytes

Freshly isolated PBMCs from patient and healthy controls were plated at 3 × 10^6^/ml density and stained for live and dead cells. For TIM-3 surface staining, the cells were treated with a Fc blocker (BD Biosciences 564,219) for 10 min at RT and for surface markers for 30 min at RT, washed, and fixed for 10 min in 4% formaldehyde (Thermo Scientific; 28,908) at RT. For intracellular TIM-3 staining, the cells were washed after dead and alive staining, permeabilized with Cytofix/Cytoperm (BD Biosciences; 554,714) for 20 min at + 4 °C, washed with cold Perm-Wash (BD Biosciences; 554,714) and treated with a Fc blocker (BD Biosciences 564,219) for 10 min at RT. Then the cells were stained with antibodies for 35 min at + 4 °C.

#### Cell Surface and Intracellular Staining of TIM-3 in PHA Stimulated T Cells

Freshly isolated PBMCs from the patient and healthy controls were plated at 1 × 10^6^/ml density in supplemented RPMI 1640 medium (2.6), allowed to rest overnight and stimulated with 1.25 µg/ml phytohemagglutinin (PHA) for 4 days. For TIM-3 surface staining, the cells were stained for live and dead cells and surface markers, washed, fixed for 10 min at RT in 4% formaldehyde (Thermo Scientific; 28,908). For intracellular TIM-3 staining, the cells were stained for live and dead cells, washed, permeabilized with Cytofix/Cytoperm (BD Biosciences; 554,714) for 20 min at + 4 °C and washed with cold Perm-Wash (BD Biosciences; 554,714). Finally, the antibodies were added and incubated for 35 min at + 4 °C.The intracellular TIM-3 median fluorescence intensity (MFI) value was calculated with the following formula: MFI TIM-3 obtained from intracellular staining – MFI TIM-3 obtained from surface staining. The TIM-3 expression was first analyzed on the surface of PHA induced CD4^+^ and CD8^+^ T lymphoblasts and further confirmed on CD56^+^ natural killer (NK) cells and CD14^+^ monocytes. All experiments included two healthy controls and all procedures were done in duplicates.

#### Expression of Checkpoint Inhibitor Receptors LAG-3, TIM-3 and PD-1

PBMCs from patient and healthy controls were plated at 2.5 × 10^5^/ml density in supplemented RPMI 1640 medium (2.6) and allowed to rest overnight. The cells were stimulated with 8 µg/ml Phytohemagglutinin (PHA-P) (Sigma Aldrich; L-1668) for 3 days. The cells were washed and stained for live and dead cells and surface markers for 30 min at RT, washed and fixed with 4% formaldehyde (Thermo Scientific; 28,908). The experiment was performed once with two healthy controls, all stained samples were done in duplicates.

#### Regulatory T Cell Analysis

PBMCs from patient and healthy controls were plated at 3 × 10^6^/ml in supplemented RPMI 1640 medium (2.6). The cells were allowed to rest overnight and stained for live and dead cells. The cells were stained for surface markers in Brilliant Stain Buffer (BD Biosciences; 563,794) for 30 min at 4 °C, washed, fixed and permeabilized with Transcription Factor Staining Buffer Set (Miltenyi Biotec; 130-122-981) for 30 min at 4 °C. After permeabilization the cells were washed and stained for FOXP3 for 35 min at 4 °C. The experiment was performed twice with two and four healthy controls, all stained samples were done in duplicates.

#### T Cell Proliferation

PBMCs from patient and healthy controls were plated at 3 × 10^6^/ml density in supplemented RPMI 1640 medium (2.6), allowed to rest overnight, washed and stained with CellTrace™ CFSE Cell Proliferation Kit (Invitrogen; C34554) for 8 min at + 37 °C. Cold supplemented RPMI was added and incubated for 10 min at RT. Then the cells were centrifuged and resuspended in RPMI medium supplemented as described above (2.6). The cells were plated at 1 × 10^6^/ml and following stimulants were added: 2.5 µg/ml or 1.25 µg/ml PHA-L solution (Invitrogen; 00-4977-93) or Anti-CD3/anti-CD28 beads (Gibco; 11131D), CD3 (Miltenyi Biotec; 130-093-387) and CD28 (Miltenyi Biotec; 130-093-375) antibodies, all stimulants were done in triplicates both with or without 100U/ml IL-2 (Peprotech; #200-02). The cells were stimulated for 4 days and stained for CD4 and CD8 surface markers before acquisition. The experiment was performed twice with two healthy controls, all stained samples were done in triplicates.

#### Intracellular Staining of Interleukin 2 (IL-2)

PBMCs from patient and healthy controls were plated 3 × 10^6^/ml density in supplemented RPMI 1640 medium (2.6) and allowed to rest overnight. A protein transport inhibitor containing Brefeldin A and Monensin (Invitrogen; 00-4980) was added to unstimulated cells or the cells were stimulated for 5 h with PMA/Cell Stimulation Cocktail with protein transport inhibitors (Invitrogen; 00-4975-93). The cells were collected, washed, and stained for live and dead cells and surface markers for 30 min at RT. The cells were washed, permeabilized with Cytofix/Cytoperm (BD Biosciences; 554,714) for 20 min at + 4 °C, washed with cold Perm-Wash (BD Biosciences; 554,714) and stained for 35 min at + 4 °C with IL-2 antibody. The experiment was performed twice with two healthy controls, all stained samples were done in duplicates.

#### Intracellular IFN-γ Staining

PBMCs from patient and healthy controls were plated at 3 × 10^6^/ml density in supplemented RPMI 1640 medium (2.6) and allowed to rest overnight. Protein transport inhibitors (Invitrogen; 00-4980) were added to unstimulated cells, or the cells were stimulated for 5 h with PMA/Cell Stimulation Cocktail with protein transport inhibitors (Invitrogen; 00-4975-93). After stimulation, the cells were collected, washed, and stained for live and dead cells and surface markers. The cells were washed twice, permeabilized with Cytofix/Cytoperm (BD Biosciences; 554,714) for 20 min at + 4 °C, washed twice with cold Perm-Wash (BD Biosciences; 554,714) and stained with IFN-γ antibody for 35 min at + 4 °C. The experiment was performed twice with two healthy controls, all stained samples were done in duplicates.

#### STAT Phosphorylation

For signal transducer and activator of transcription (STAT) 1 and 3 previously described methods were used [16, 17]. For STAT4, PBMCs from patient and healthy controls were plated at 4 × 10^6^/ml density in supplemented RPMI 1640 medium (2.6) and allowed to rest overnight. The cells were subjected to a 3-day pre-stimulation with 1.25 µg/ml PHA-L (Invitrogen; 00-4977) and 100U/ml IL-2 (Peprotech; #200-02), collected, washed, and stained for live and dead cells. The cells were washed, resuspended in medium and allowed to rest for one hour. The cells were stimulated with 10ng/ml recombinant IL-12 (Peprotech; #200 − 12) for 20 min at 37 °C and immediately fixed 10 min in 4% formaldehyde (Thermo Scientific; 28,908) at RT. After two washes, the cells were permeabilized with ice cold Perm Buffer III (BD Biosciences; 558,050) for 30 min on ice, washed and stained for 35 min at + 4 °C with antibodies. The STAT4 experiment was performed twice with two healthy controls, all stained samples were done in duplicates.

## Results

### Clinical Description

**Fig. 1:**
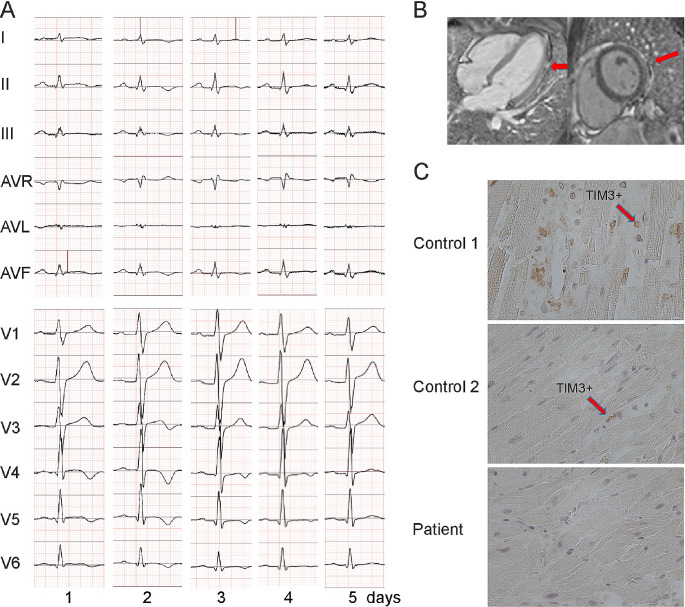
(**A**) Representative electrocardiographs (ECG, 50 mm/s) on days 1 to 5 of hospitalization during an episode of acute myocarditis at age 17 years (**B**) Late gadolinium enhancement pattern (arrows) consistent with myocarditis in cardiac MRI (**C**) Representative images of TIM-3 immunostaining in myocardium biopsies. TIM-3 staining was positive (red arrows) in the control and negative in the patient’s samples

A male patient with non-consanguineous parents was healthy until the age of 4 years when he started to develop episodes of fever and chest discomfort. At the initial presentation, electrocardiography (ECG) showed sinus tachycardia with T-wave inversions and his C-reactive protein (CRP) was elevated (57 mg/L). He was concluded to suffer from mild myocarditis from which he recovered well. At the age of 5 years, he experienced another episode of chest pain and sinus tachycardia with T-wave inversions. CRP (58 mg/L) and troponin I (TnI) (2.1 ng/mL; normal < 0.03 ng/mL) were elevated. Following these first episodes, his growth, physical abilities, and developmental path continued to progress despite the recurrent myocarditis episodes. He received a diagnosis of allergic asthma and underwent treatment for it at the age of 9. He continued to suffer recurrent febrile episodes accompanied by chest pain, tachycardia, an elevated troponin T (TnT) and T-wave inversions in his ECG. Representative ECG findings consistent with myocarditis episode (TnT 485 ng/L, normal < 50 mg/L) at the age of 17 years are shown in Fig. 1A. During that episode, echocardiography showed a mild and transient reduction in left ventricular function. Myocardial magnetic resonance imaging (MRI) (Fig. 1B) demonstrated edema and enhancement consistent with myocarditis. Cardiac muscle antibodies were negative, and no evidence of respiratory infection pathogens was found (Supplemental Methods). The patient did not develop clinical or laboratory findings suggestive of HLH [18] Histological staining of myocardial biopsies collected from four locations were positive for CD3^+^ T cells (14/mm^2^) and macrophages consistent with myocarditis [9]. The myocardial biopsy was negative for TIM-3 expression (Fig. 1C). Beginning at the age of 17 years, febrile myocarditis episodes were treated with anakinra, an IL-1β receptor antagonist (Kineret®, 100 mg daily, 4 to 7 days), with a good clinical response. Due to successful anakinra response, the patient has not required hospitalization. Despite the tonsillectomy due to episodes of tonsillitis, he has not encountered additional health concerns.

### Genetics

WES (Blueprint Genetics, Helsinki, Finland) found the patient to be homozygous for the pathogenic HAVCR2 gene c.245 A > G p.Tyr82Cys variant (allele frequency in Finnish population 0.004737) [5]. The patient was also found to be a heterozygous asymptomatic carrier of 47.74 kb in-frame duplication in PRKDC gene. No other genetic variants affecting immunity were found.

### TIM-3 Surface Expression is Negative in p.Tyr82Cys Variant Cells

**Fig. 2:**
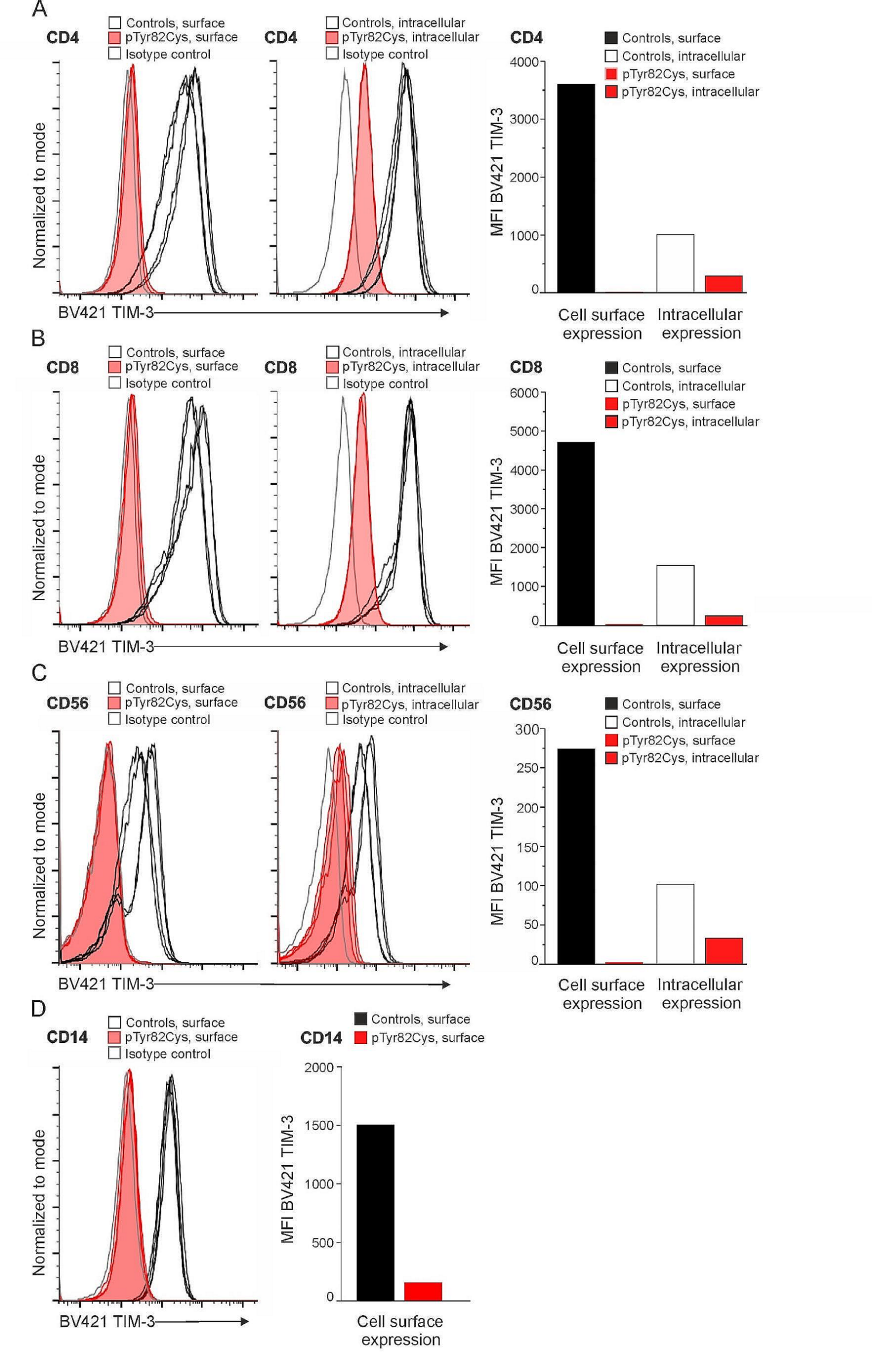
(**A**) TIM-3 surface and intracellular expression in CD4^+^ T cells. Graph shows quantified mean fluorescence index (MFI) values for cell surface and intracellular TIM-3 expression. (**B**) TIM-3 surface and intracellular expression in CD8^+^ T cells. Graph shows quantified MFI values for cell surface and intracellular TIM-3 expression. (**C**) TIM-3 surface and intracellular expression in NK cells. Graph shows quantified MFI values for cell surface and intracellular TIM-3 expression. (**D**) TIM-3 surface expression in CD14^+^ monocytes with a graph showing quantified MFI values for surface TIM-3 expression

PBMCs were collected and analyzed from the patient when he was asymptomatic. First, we found that the PHA induced CD4^+^ (Fig. 2A) and CD8^+^ (Fig. 2B) T lymphoblasts were negative for TIM-3 surface expression. Second, the CD56^+^ natural killer (NK) (Fig. 2C) cells and CD14^+^ monocytes (Fig. 2D) were also confirmed to be negative for TIM-3 surface expression. Further, intracellular TIM-3 staining was low in the CD4^+^ and CD8^+^ T lymphoblasts and CD56^+^ NK cells when compared to healthy controls (Fig. 2A-C). No evidence of intracellular TIM-3 accumulation was seen.

**Fig. 3:**
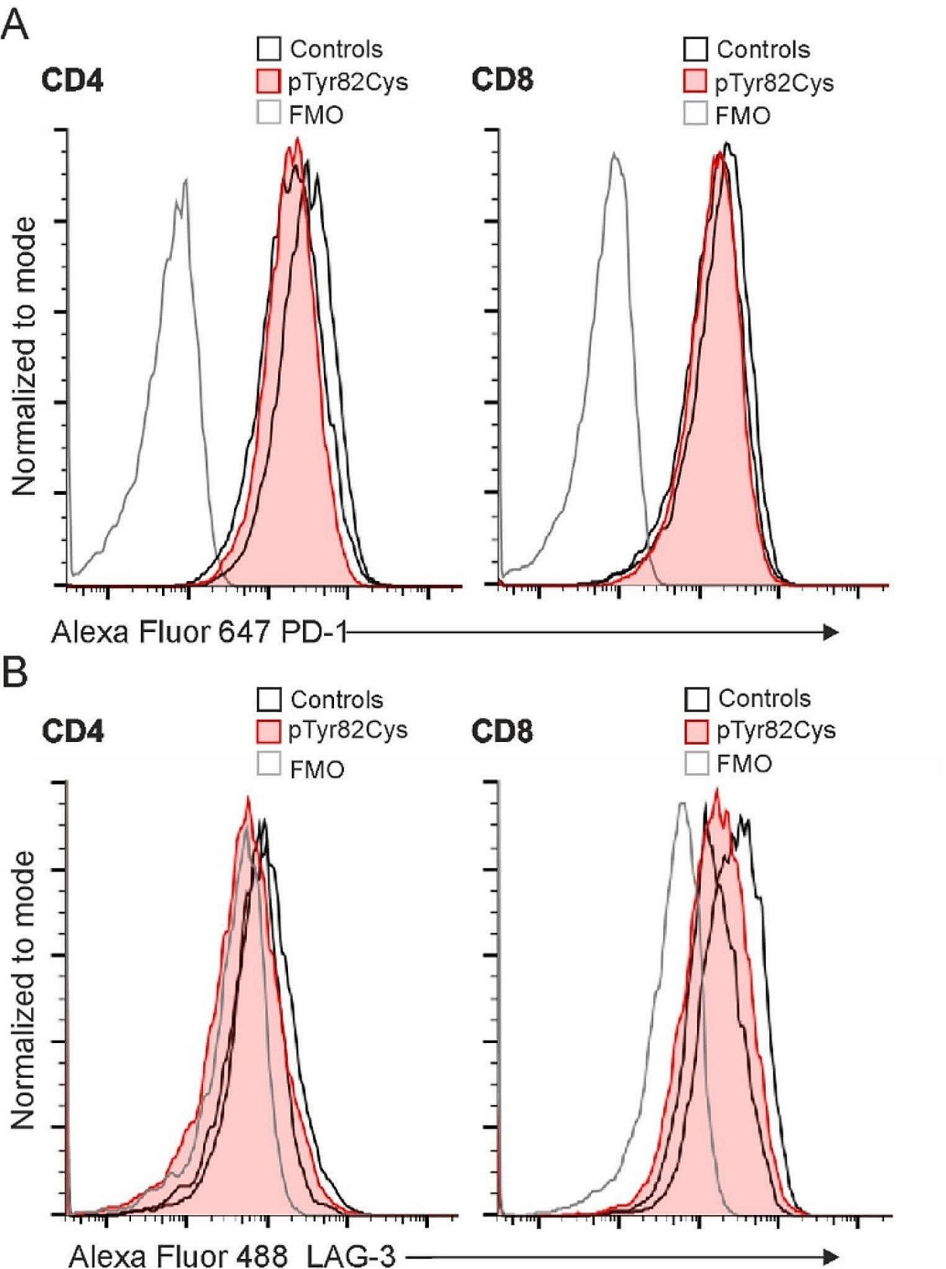
(**A**) PD-1 expression in 3-day PHA stimulated CD4^+^ and CD8^+^ T lymphoblasts. (**B**) LAG-3 expression in 3-day PHA stimulated CD4^+^ and CD8^+^ T lymphoblasts

Expression of programmed cell death protein 1 (PD-1) and lymphocyte-activation gene 3 (LAG-3) were analyzed in TIM-3 p.Tyr82Cys T lymphoblasts since several checkpoint receptors are co-expressed with TIM-3. Expressions of PD-1 (Fig. 3A) and LAG-3 (Fig. 3B) in CD4^+^ and CD8^+^ T cells after stimulation with PHA for 3-days were comparable to the controls.

### Distribution of Peripheral Blood Lymphocytes is Normal

Distribution of B and T cell populations and their subclasses were mainly unaffected although the number of Treg cells (CD4^+^CD25^+^CD127^−^) and the percentage of recent thymic emigrant T cells were slightly elevated (Table 1). Proportions of naïve and effector/memory Treg populations as well as Treg subpopulations positive for CCR7, CCR6 and CXCR3 were comparable to controls (data not shown). Proportions of T helper 1 (Th1) (CD4^+^CD45RA^−^CXCR3^+^) and 17 (Th17) (CD4^+^CD45RA^−^CCR6^+^) cells were also unaffected. These findings agree with the role for TIM-3 at late phase of differentiation of activated effector T cells (Th1/Tc1); TIM-3 deficiency does not significantly affect the proportions of circulating T cell populations.

**Table 1:** Distribution of B and T cell populations and their subclasses in peripheral blood of the index patient homozygous for TIM-3 p.Tyr82Cys. Blood samples were collected when the patient was asymptomatic

Leukocytes			Normal range
	Lymphocytes	1.6 × 10^9^/L	1.2–3.5 × 10^9^/L
	Monocytes	0.9 × 10^9^/L	0.2–0.8 × 10^9^/L
	Neutrophils	4.5 × 10^9^/L	1.6–6.3 × 10^9^/L
	Basophils	0.02 × 10^9^/L	< 0.09 × 10^9^/L
	Platelets	275 × 10^9^/L	150–360 × 10^9^/L
	NK cells	196 × 10^6^/L	84–724 × 10^6^/L
**CD19** ^**+**^ **B cells**		254 × 10^6^/L	80–616 × 10^6^/L
Transitional	CD38^++^IgM^+^	3.6%↑	0.6–3.5%
Naive	CD27^−^IgD^+^IgM+	73.6%	43.2–82.4%
Memory	CD27^+^	23.8%	
Marginal zone	CD27^++^IgD^+^IgM^+^	13.5%	7.2–30.8%
Switched memory	CD27^+^IgD^−^IgM^−^	7.4%	6.5–29.2%
Plasmablasts	CD38^++^IgM^−^	0.4%	0.4–3.6%
Activated	CD38^low^CD21^low^	6.8%	0.8–7.7%
	S-IgA	2.60	0.88-4.84 g/L
	S-IgG	13.3	6.77-15.0 g/L
	S-IgM	0.82	0.36-2.59 g/L
	S-IgE	543	< 160IU/L
**CD3** ^**+**^ **T cells**		1181 × 10^6^/L	742–2750 × 10^6^/L
	CD45RA^+^CD62L^+^CD31^+^	40.4%↑	14.4–38.3%
	TCRαβ^+^	93.8%	88.1–97.8%
	TCRγδ^+^	6.2%	1.9–11.7%
	CD4^−^CD8^−^ TCRαβ^+^	1.2%	
**CD3** ^**+**^ **CD4** ^**+**^ **T cells**		716 × 10^6^/L	404–1612 × 10^6^/L
TCM	CCR7^+^CD45RA-	35.7%↑	8.4–32.8%
Naive	CCR7^+^CD45RA^+^	51.4%	20.5–54.8%
TEM	CCR7^−^CD45RA^−^	12.3%↓	19.9–52.4%
Temra	CCR7^−^CD45RA^+^	0.6%↓	1.4–17.0%
Th17		21.8%	13–34%
Th1		25.8%	16–32%
Treg	CD25^+^CD127^low^	6.5%	3.7–5.6% (*n* = 4)
**CD3** ^**+**^ **CD8** ^**+**^ **T cells**			
TCM	CD45RA^−^CCR7^+^	9.2%	8.4–32.8%
Naïve	CD45RA^+^CCR7^+^	58.0%↑	20.5–54.8%
TEM	CD45RA^−^CCR7^−^	21.0%	19.9–52.4%
Temra	CD45RA^+^CCR7^−^	11.9%	1.4–17.0%

### IL-1β, INF-γ and STAT Responses

**Fig. 4:**
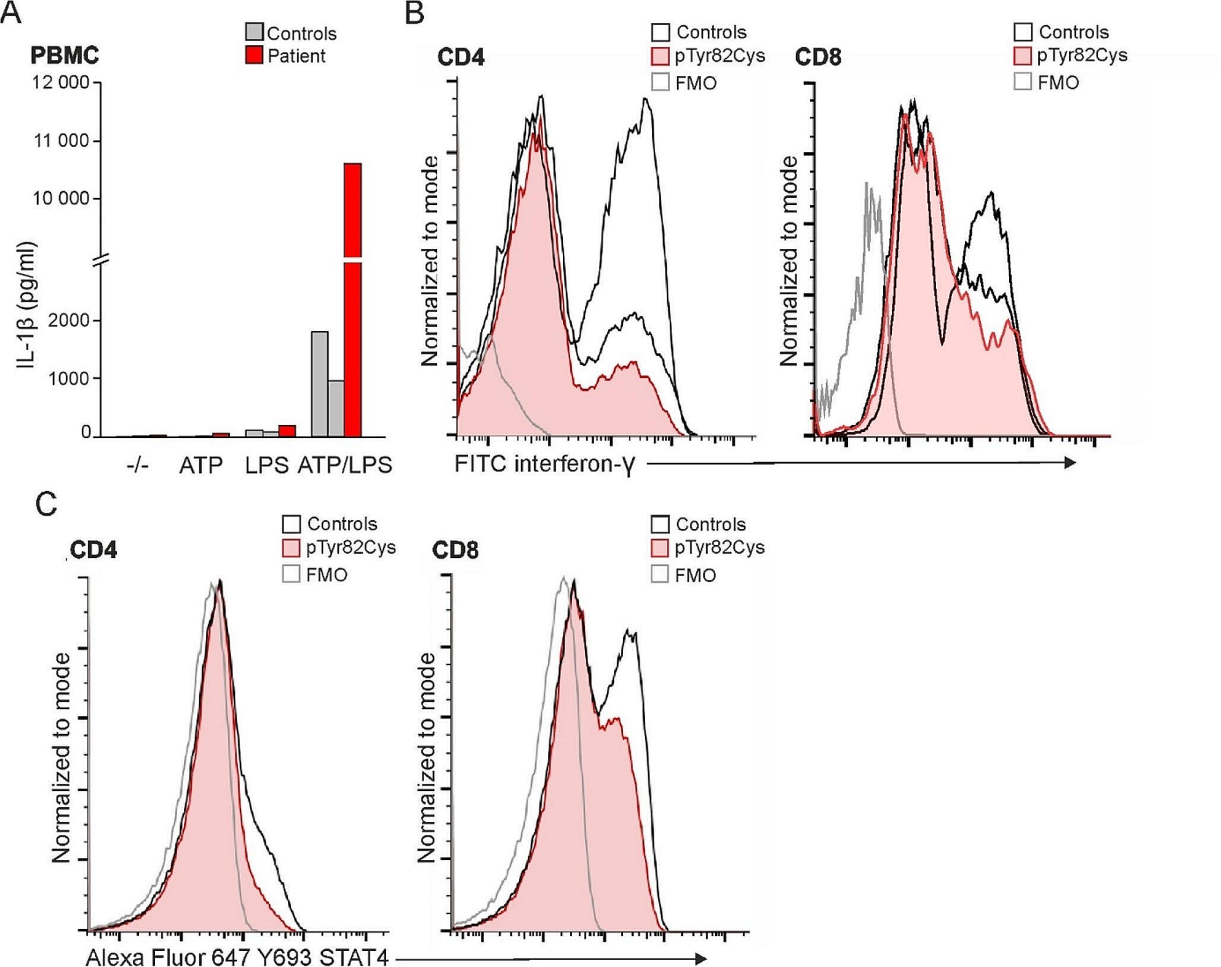
(**A**) IL-1β secretion was measured in LPS/ATP stimulated PBMCs of p.Tyr82Cys index and healthy controls. (**B**) Interferon-γ production was measured in 5-hour PMA stimulated CD4^+^ and CD8^+^ T cells. (**C**) STAT4 phosphorylation determined by flow cytometry; PHA and IL-2 pre-stimulated CD4^+^ and CD8^+^ T lymphoblasts were activated with IL-12 for STAT4 phosphorylation

Elevation in IL-1β was observed in patient’s LPS-primed and ATP-stimulated PBMCs (Fig. 4A). Clinical response to anakinra treatment during myocarditis was consistent with the IL-1β overproduction. Serum IL-6 (3.5ng/L) was not elevated during myocarditis episode or when the patient was asymptomatic (normal < 7ng/L). IFN-γ production was not excessive in PMA-stimulated CD4^+^ and CD8^+^ T cells (Fig. 4B). The phosphorylation of STAT4, which is required for the IL-12 induced IFN-γ production, was not overly activated after PHA induction in CD4^+^ and CD8^+^ T lymphoblasts when compared with controls (Fig. 4C). Phosphorylation of STAT1 and STAT3, potential mediators of IFN-γ production [19], were also normal when compared to healthy controls (data not shown). These findings are consistent with the lack of inflammatory symptoms or other health complaints by the patient in between the myocarditis episodes.

### TIM-3 p.Tyr82Cys T Cell Proliferation

**Fig. 5:**
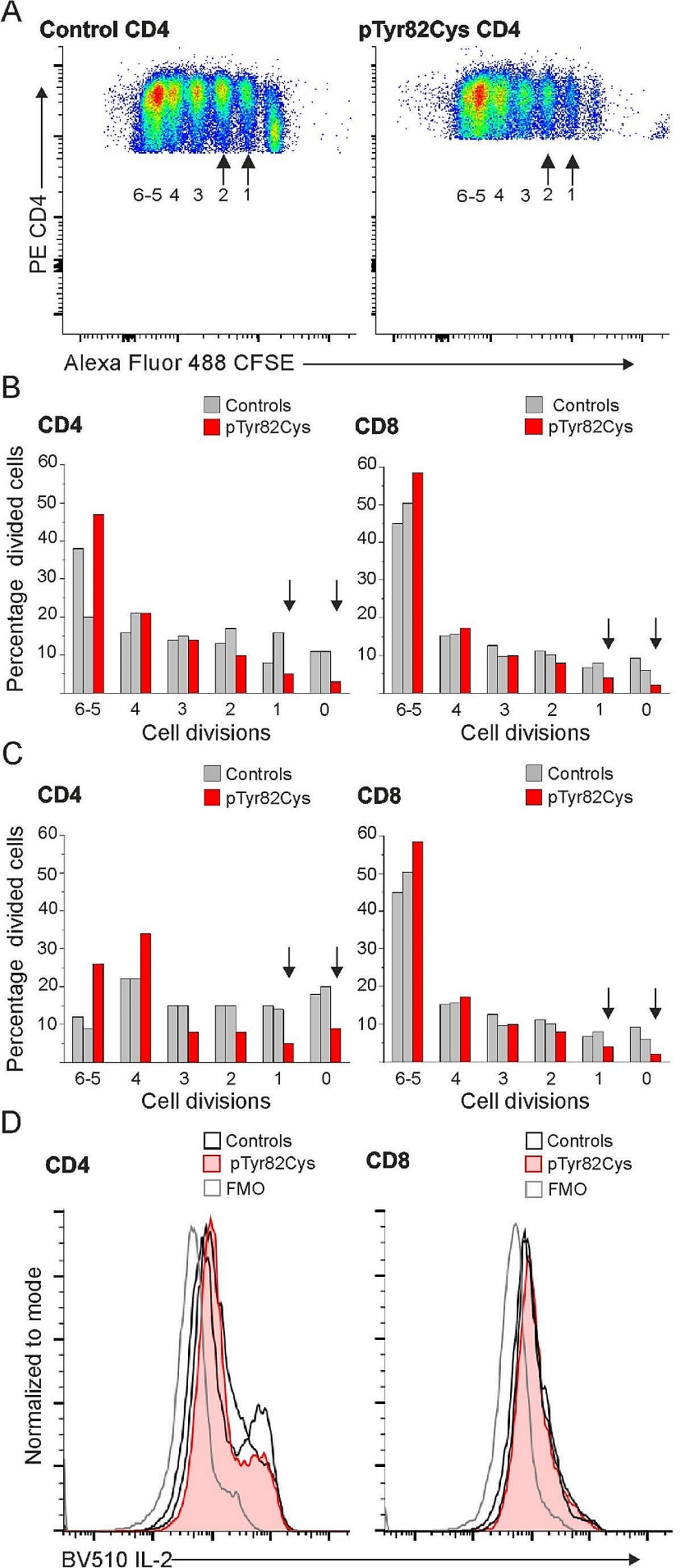
(**A**) Proliferation in CD4^+^ T lymphoblasts was determined by flow cytometry after 4-day stimulation with CD3/CD28 antibodies. (**B**) Proliferated populations of CD4^+^ and CD8^+^ T lymphoblasts after 4-day stimulation with CD3/CD28 antibodies (**C**) Proliferated populations of CD4^+^ and CD8^+^ T lymphoblasts after 4-day PHA stimulation. (**D**) Interleukin-2 (IL-2) production in CD4^+^ and CD8^+^ T cells after five-hour PMA stimulation

T cell proliferation in our TIM-3 deficient index was active; a high proportion of T cells underwent four to six divisions after stimulus with PHA, CD3/CD28 beads or immobilized CD3 antibodies/soluble CD28 antibodies, with or without IL-2 stimulus when compared to control cells (Fig. 5). The populations of stimulated CD4^+^ T lymphoblasts that had divided only once or twice were decreased when compared with healthy controls (Fig. 5A and B). A similar result was seen in CD3/CD28 antibody-stimulated CD8^+^ T lymphoblasts (Fig. 5B) as well as PHA-stimulated CD4^+^ and CD8^+^ T lymphoblasts (Fig. 5C). IL-2 expression in vitro was comparable to that observed in healthy control CD4^+^ and CD8^+^ T cells after a five-hour stimulation with PMA (Fig. 5D). This result suggests that the increased T cell proliferation in the case described here can not be explained by increased IL-2 production.

## Discussion

A growing number of monogenic autoinflammatory syndromes characterized by systemic inflammation have been found [7, 20, 21]. While autoinflammatory patients commonly suffer from rashes, pain in muscles, joints or abdomen, the cardiovascular system can also be affected; recurrent inflammatory pericarditis, secondary myocardial amyloidosis or early atherosclerosis due to autoinflammation have been described [13, 14]. Patients with familial mediterranean fever (FMF), for example, may suffer from recurrent pericarditis episodes and FMF patients with inadequate treatment may develop myocardial amyloidosis [21]. Genetic mechanisms associated with recurrent myocarditis episodes, however, are not well understood [9]. The patient described here suffers from a life-long history of early onset and recurrent myocarditis episodes confirmed with clinical, radiological and histological findings. Based on the clinical history, it is reasonable to assume that genetic and biological defects in the regulation of inflammatory responses can be implicated in this rare condition.

The role of TIM-3 deficiency, high IL-1β production and hyperinflammation were previously studied among patients suffering from SPTCL, a rare form of T cell lymphoma, which can be complicated by HLH [5]. While SPTCL can be indolent, some TIM-3 deficient SPTCL patients develop hyperinflammatory HLH [22]. HLH, a sepsis-like condition with multiorgan involvement and high mortality, can also seriously affect the circulation and the heart [18, 23]. Clinical significance of TIM-3 deficiency is further demonstrated among patients who receive immune checkpoint inhibitor treatments; they may also develop myocarditis [24]. Moreover, understanding of TIM-3 deficiency is provided by animal studies; a reduction in TIM-3 signaling can lead to myocardial inflammation in an experimental mouse model [3]. These previous observations support the view that TIM-3 deficiency can contribute not only to hyperinflammation but also to development of myocarditis [1]. Although our experience is based on one patient and the role of additional genetic mechanisms cannot be excluded, it seems possible that the TIM-3 deficiency contributes to inflammatory features observed in our patient.

The blood cells were collected and extensively analyzed when the patient was asymptomatic in between the febrile myocarditis episodes. Importantly, the results obtained in this report are not affected by lymphoma or HLH. De Luca et al. and Van Den Eeckhout et al. suggested that development of myocarditis may depend on T cell proliferation and high IL-1β production [25, 26]. Consistent with these, we showed an intense IL-1β response in vitro and an unrestrained proliferation of TIM-3 deficient T cells collected from our patient. These features may share biological mechanisms with SPTCL and may also contribute to T cell positive histological findings in myocardial biopsy. In summary, we hypothesize that lack of TIM-3 inhibitory effects allows high IL-1β production in monocytes which in turn stimulates TIM-3 deficient T cells to proliferate and migrate. Lack of excessive IL-2, IL-6 or IFN-γ responses in our TIM-3 deficient patient highlights the view that immunological presentations in SPTCL/HLH or primary HLH are different from the myocarditis case described here [5]. 

The genetics and biology of myocarditis are not well understood; our findings create novel understanding on biological mechanisms associated with myocarditis and cardiovascular manifestations of autoinflammatory conditions [13, 27]. Our experience may also bring understanding on myocarditis caused by immune checkpoint inhibitor treatments [24]. Importantly, a very positive anakinra clinical response supports the view that the role of IL-1 inhibition should be considered in other patient populations with myocarditis [28–30]. In summary, we conclude that the possibility of genetic TIM-3 deficiency should be evaluated whenever recurrent myocarditis is suspected [8, 9]. 

### Electronic Supplementary Material

Below is the link to the electronic supplementary material.

Supplementary Material 1

## Data Availability

The datasets generated during and/or analyzed during the current study are available from the corresponding author on reasonable request.
