# Supplementary material for: Germline HAVCR2/TIM-3 Checkpoint Inhibitor Receptor Deficiency in Recurrent Autoinflammatory Myocarditis
Source: J Clin Immunol. 2024 Mar 15;44(3):81. doi: 10.1007/s10875-024-01685-x (PMC10940375; doi:10.1007/s10875-024-01685-x)
Supplement: Supplementary file 1 — Supplementary Material 1 [file 10875_2024_1685_MOESM1_ESM.docx]

**Supplementary materials**

**Germline HAVCR2/TIM-3 checkpoint inhibitor receptor deficiency in recurrent autoinflammatory myocarditis**

Nora Pernaa^a^, Anni Vakkuri^a^, Miika Arvonen^b^, Outi Kuismin^c^, Wenny Santaniemi^a^, Virpi Glumoff^d^, Elisa Lappi-Blanco^e^, Ulla Lantto^f^ , Marjo Okkonen^a^, Kari Kaikkonen^a^, Juhani Junttila^a g^, Risto Kerkelä^a g^, Pirjo Åström^a g^, Timo Hautala^a h^

**Supplementary methods**

**Antibodies used in the flow cytometry stainings:**

**Cell surface and intracellular staining of TIM-3 in NK cells and monocytes**

Antibodies used in the staining: PE Cy7 anti-human CD56 clone B159 (BD Biosciences; 557747), PE CF594 anti-human CD14 clone MφP9 (BD Biosciences; 562335), BV421 anti-human TIM-3/CD366 clone 344823 (BD Biosciences; 747962), BV421 Rat IgG2a κ Isotype Control clone R35-95 (BD Biosciences; 562602).

**Cell surface and intracellular staining of TIM-3 in PHA stimulated T cells**

Antibodies used in the staining: FITC anti-human CD3 clone SK7 (345764), APC anti-human CD8 clone RPA-T8 (BD Biosciences; 555369) and BV421 anti-human TIM-3/CD366 clone 344823 (BD Biosciences; 747962), BV421 Rat IgG2a κ Isotype Control clone R35-95 (BD Biosciences; 562602).

**Expression of checkpoint inhibitor receptors LAG-3, TIM-3 and PD-1**

Antibodies used in the staining: BV421 anti-human TIM-3/CD366 clone 344823 (BD Biosciences; 747962), BV421 Rat IgG2a κ Isotype Control clone R35-95 (BD Biosciences; 562602), Alexa Fluor 488 anti-human LAG3/CD223 clone T47-530 (BD Biosciences; 567610), Alexa Fluor 647 anti-human PD-1/CD279 clone MIH4 (BD Biosciences; 566850), PE Cy7 anti-human CD4 clone SK3 (BD Biosciences; 557852), APC anti-human CD8 clone RPA-T8 (BD Biosciences; 555369), Alexa fluor 488 anti-human CD3 clone UCHT1 (BD Biosciences; 557694) and PE anti-human CD3 clone UCHT1 (BD Biosciences; 555333).

**Regulatory T cell analysis**

The antibodies used in the staining: BV510 anti-human CD4 clone SK3 (BD Biosciences; 562970), PE Cy7 anti-human CD127 clone HIL-7R-M21 (BD Biosciences; 560822), APC anti-human CD45RA clone 5H9 (BD Biosciences; 561210), BV421 anti-human CD25 clone 2A3 (BD Biosciences; 564033), PE anti-human CCR7 clone 2-L1-A (BD Biosciences; 566741) or PE anti-human CCR6 clone 11A9 (BD Biosciences; 551773) or PE anti-human CXCR3 clone 1C6/CXCR3 (BD Biosciences; 550633). The Alexa Fluor 488 anti-human FOXP3 clone 206D (#320112) was purchased from Biolegend.

**T cell proliferation**

Antibodies used in the staining: PE anti-human CD4 clone RPA-T4 (BD Biosciences; 561844) and APC anti-human CD8 clone RPA-T8 (BD Biosciences; 561953).

**Intracellular staining of interleukin 2 (IL-2)**

Antibodies used in the staining: PE Cy7 anti-human CD4 clone SK3 (BD Biosciences; 557852), APC anti-human CD8 clone RPA-T8 (BD Biosciences; 555369), FITC anti-human CD3 clone SK7 (BD Biosciences; 345764) and BV510 anti-human IL-2 clone 5344.111(RUO) (BD Biosciences; 564365).

**Intracellular staining of IFN-γ after 5-hour PMA stimulation**

Antibodies used in the staining: PE Cy7 anti-human CD4 clone SK3 (BD Biosciences; 557852), APC anti-human CD8 clone RPA-T8 (BD Biosciences; 555369) and PE CF594 anti-human CD3 clone SP34-2 (BD Biosciences; 562406). FITC anti-human interferon-γ clone 4S.B3 (Invitrogen;11-7319-82).

**STAT4 phosphorylation**

Antibodies used in the staining: FITC anti-human CD3 clone SK7 (BD Biosciences; 345764), PerCP eFluor 710 anti-human CD4 clone SK3 (Invitrogen; 46-0047-42) or PE Cy7 anti-human CD4 clone SK3 (BD Biosciences; 557852) and Alexa Fluor 647 anti-human p693 STAT4 clone 38/p-Stat4 (BD Biosciences; 558137).

**Respiratory virus nucleic acid analysis**

Infectious agents were analyzed from nasal swab samples with accredited nucleic acid amplification methods at Nordlab Oulu laboratory.

(<https://tutkimusohjekirja.nordlab.fi/ohjekirja/nayta.tmpl?sivu_id=146&setid=8818>)

Adenovirus (-AdenNhO)

Bocavirus (-BocaNhO)

Coronavirus 229E (-Kor229E)

Coronavirus HKU1 (-KorHKU1)

Coronavirus NL63 (-KorNL63)

Coronavirus OC43 (-KorOC43)

Metapneumovirus (-hMPVNhO)

Influenza A (-InfANhO)

Influenza A/H1 (-InfAH1)

Influenza A/H1-2009 (-infAH09)

Influenza A/H3 (-InfAH3)

Influenza B (-InfBNhO)

Parainfluenza 1 (-Pin1NhO)

Parainfluenza 2 (-Pin2NhO)

Parainfluenza 3 (-Pin3NhO)

Parainfluenza 4 (-Pin4NhO)

RSV (-RSVNhO)

Rhinovirus/Enterovirus (-RiEnNhO)

Sars-CoV-2-virus (-CV19NhO)

**Respiratory bacterial nucleic acid analysis**

Bordetella pertussis (-BopeNhO)

Legionella pneumophila (-LepnNhO)

Mycoplasma pneumoniae (-MypnNhO)
